# Astral Microtubule Pivoting Promotes Their Search for Cortical Anchor Sites during Mitosis in Budding Yeast

**DOI:** 10.1371/journal.pone.0093781

**Published:** 2014-04-10

**Authors:** Stephan Baumgärtner, Iva M. Tolić

**Affiliations:** 1 Max Planck Institute of Molecular Cell Biology and Genetics, Dresden, Germany; 2 Division of Molecular Biology, Ruđer Bošković Institute, Zagreb, Croatia; Fondazione Edmund Mach, Research and Innovation Centre, Italy

## Abstract

Positioning of the mitotic spindle is crucial for proper cell division. In the budding yeast *Saccharomyces cerevisiae*, two mechanisms contribute to spindle positioning. In the Kar9 pathway, astral microtubules emanating from the daughter-bound spindle pole body interact via the linker protein Kar9 with the myosin Myo2, which moves the microtubule along the actin cables towards the neck. In the dynein pathway, astral microtubules off-load dynein onto the cortical anchor protein Num1, which is followed by dynein pulling on the spindle. Yet, the mechanism by which microtubules target cortical anchor sites is unknown. Here we quantify the pivoting motion of astral microtubules around the spindle pole bodies, which occurs during spindle translocation towards the neck and through the neck. We show that this pivoting is largely driven by the Kar9 pathway. The microtubules emanating from the daughter-bound spindle pole body pivot faster than those at the mother-bound spindle pole body. The Kar9 pathway reduces the time needed for an astral microtubule inside the daughter cell to start pulling on the spindle. Thus, we propose a new role for microtubule pivoting: By pivoting around the spindle pole body, microtubules explore the space laterally, which helps them search for cortical anchor sites in the context of spindle positioning in budding yeast.

## Introduction

Proper segregation of genetic material between the two daughter cells requires the mitotic spindle to become properly positioned with respect to the cell division plane. In addition to controlling chromosome segregation, the position of the spindle determines the fate of the daughter cells during development of metazoan organisms. Forces that orient and position the spindle are typically generated at the sites where astral microtubules, which grow from the centrosomes, interact with the cell cortex.

A large body of knowledge about the general principles governing spindle positioning comes from studies in the budding yeast *Saccharomyces cerevisiae* (reviewed in [1], [2]). As the name suggests, budding yeast divides by budding, which means that a daughter cell grows as a bud on the mother cell. The two cells are connected by a small opening called neck, through which one end of the spindle has to be moved during mitosis.

Spindle positioning and movement is driven through interactions between the plus end of astral microtubules extending from the spindle pole body (SPB, a centrosome equivalent) and the cell cortex. Two pathways have been described: (i) the Kar9 pathway is responsible for positioning the spindle close to the neck prior to anaphase [3], and (ii) the dynein pathway generates forces to pull the spindle through the neck into the daughter cell during anaphase [4], [5], [6], [7] and contributes to spindle elongation [8], [9]. In the Kar9 pathway, astral microtubule plus ends connect to cortical actin via the plus-end binding protein Bim1/EB-1, which binds to the linker protein Kar9 [10], which in turn binds to a type-V myosin Myo2 [11], [12]. Myosin walks along cortical actin filaments, thereby moving the microtubule plus end towards the neck. As the plus end moves along the cortex, the whole astral microtubule pivots around the SPB, ending up oriented towards the neck [13]. This reorientation or angular movement of microtubules requires actin, Myo2 and Kar9 [14]. Kar9 is preferentially localized at the daughter-bound SPB and its astral microtubules, thus only the microtubules extending from the daughter-bound SPB become oriented towards the neck [14]. Once at the neck, the microtubule plus end is captured by Bud6, a protein that binds actin and formin [15], which stabilizes the position of the microtubule and of the spindle near the neck.

In the dynein pathway, the plus end of a growing microtubule accumulates dynein in a Bik1/CLIP-170- and Pac1/LIS1-dependent manner. Dynein reaches the plus end by being transported along the microtubule by the kinesin Kip2 or by directly binding from the cytoplasm to the plus end [16], [17], [18], [19], but dynein may also diffuse along the microtubule, as in fission yeast [20], [21]. When the plus end brings dynein close to the cortical anchor protein Num1, dynein binds to the anchor in a process termed off-loading [19], [22], and may detach from the anchor in response to load forces, as shown in fission yeast [23]. Upon binding to the anchor, dynein starts to walk towards the minus end of the microtubule, thereby pulling on the microtubule and moving the spindle. However, for dynein to exert force to translocate the spindle, the microtubule, which carries dynein, must find anchor proteins at the cortex to off-load dynein onto the anchor. The mechanism by which microtubules target cortical anchor sites has remained unknown so far.

We have recently shown that during mitosis in fission yeast microtubules pivot around the SPB, which accelerates their search for kinetochores [24]. The pivoting motion allows microtubules to explore space laterally, as they search for targets such as kinetochores. Here we quantify the pivoting of astral microtubules in budding yeast. We propose that, similarly to the search for kinetochores, microtubule pivoting helps them to search for cortical anchor sites in order to move the spindle into the bud.

## Results

We set out to study microtubule and spindle movements by imaging budding yeast cells expressing α-tubulin-GFP with high time resolution (0.4–0.6 s). The presence of a small number of microtubules in these cells enabled us to observe the dynamics and movement of each astral microtubule during its lifetime. In order to test the role of the Kar9 pathway on these movements, we used a strain lacking Kar9, in which microtubules do not interact with actin and myosin. Likewise, to test the role of the dynein pathway, we used a strain lacking the anchor protein Num1, in which microtubules do not off-load dynein to the cortex because the cortical anchor proteins are missing.

Cells lacking Kar9 (*kar9*Δ) did not show a defect in spindle translocation into the bud, whereas the cells lacking the dynein pathway (*num1*Δ) did (Table S1), suggesting that the spindle translocation into the bud is mainly driven by dynein, consistent with previous work [4]. Astral microtubules in *kar9*Δ cells lived for significantly longer time and grew to a larger length than those in wild type, whereas the effect of deleting Num1 on microtubule dynamics was smaller (Table S2). These data suggest that Kar9 has a major effect on microtubule dynamics, consistent with a previous study [25].

### Astral Microtubules Pivot around the Spindle Pole

**Figure 1:**
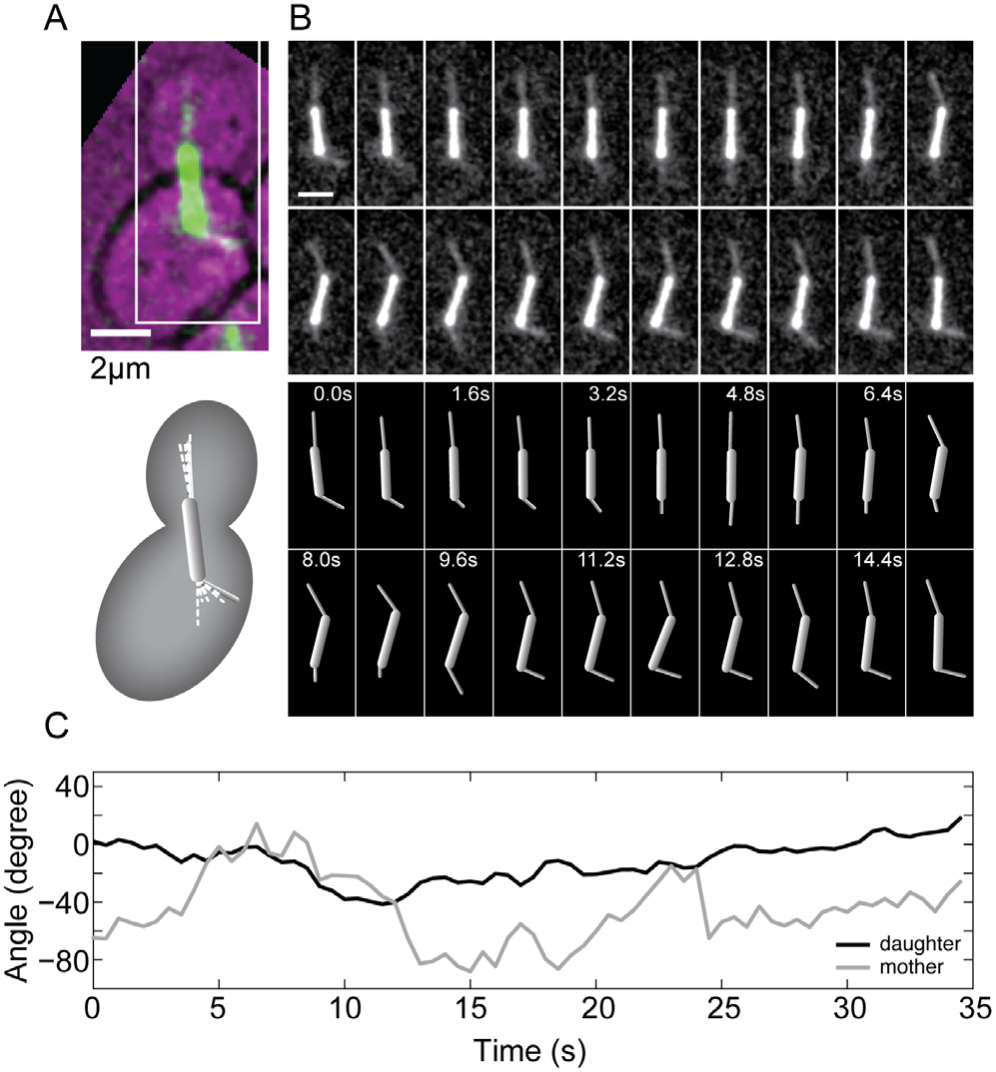
Astral microtubules pivot around the spindle pole body. (A) Image of the spindle and astral microtubules (green) in a budding yeast cell expressing tubulin-GFP (bright-field image of the cell is overlaid in magenta); scale bar is 2 μm. In the corresponding scheme below, the gray rod is the spindle and the dashed white lines represent different positions of astral microtubules. (B) Time-lapse images of the region marked by the white rectangle in panel A; scale bar is 2 μm. The schemes below show the position of the spindle and astral microtubules in each image; time is given in seconds. Note that the astral microtubules perform angular motion around the SPB, see also Movie S1. (C) Angle of the astral microtubule extending from the SPB in the daughter cell (black) and of the astral microtubule extending from the SPB in the mother cell (gray) as a function of time, for the cell shown in panels A and B. The angles were calculated with respect to the spindle.

**Figure 2:**
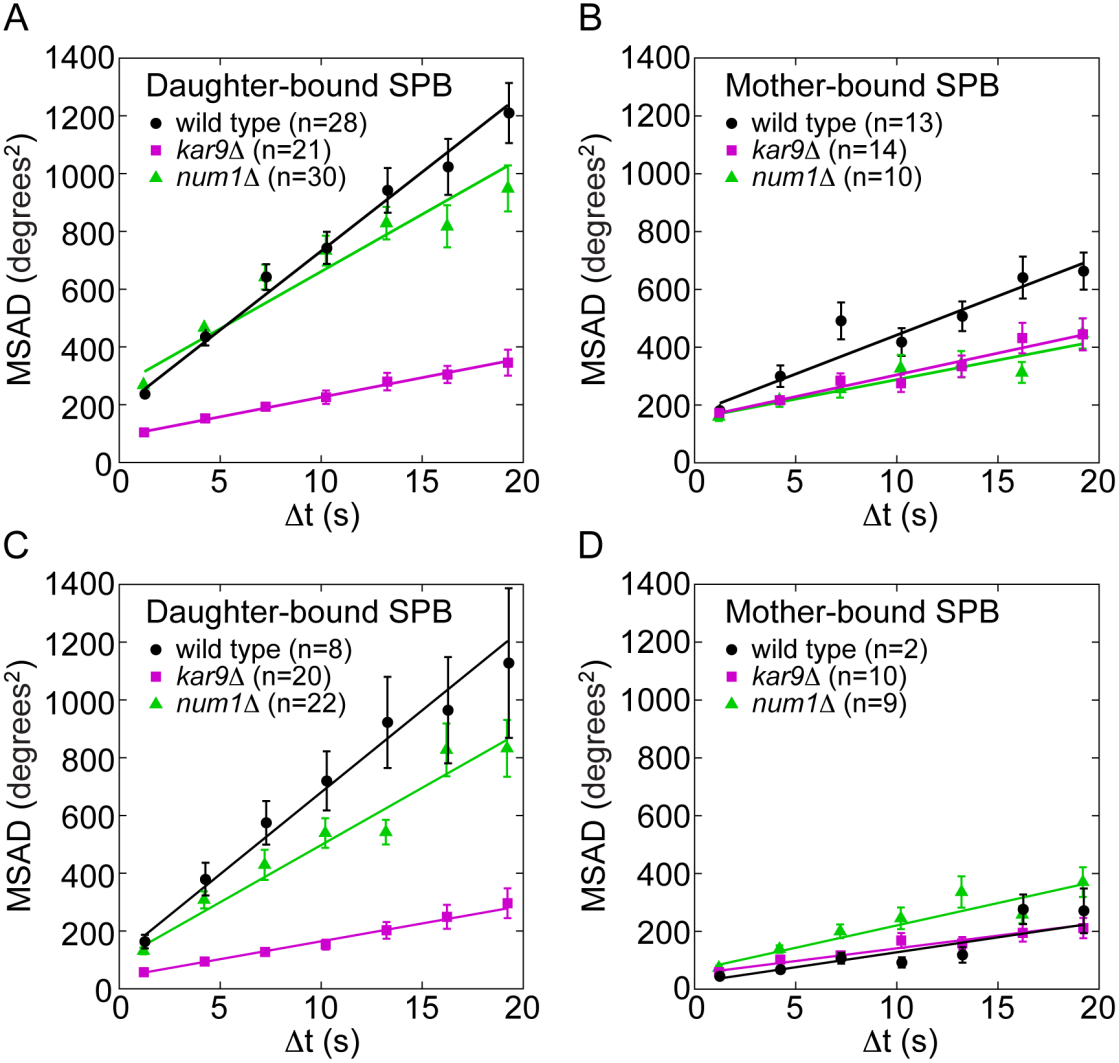
Microtubules pivot faster in cells with an intact Kar9 pathway. Mean squared angular displacement (MSAD) of astral microtubules extending from the SPB destined to the daughter (A and C) and from the SPB destined to the mother cell (B and D). In A and B, microtubules of length 0.75–1.25 μm were included, whereas in C and D, microtubules of length 1.25–1.75 μm were included in the analysis. Wild-type cells are shown in black, *kar9*Δ in magenta, and *num1*Δ in green. One-minute-long time series of the angle were used, error bars represent s.e.m., n denotes the number of microtubules. The number of cells was 9 for wild type, 10 for *kar9*Δ and 10 for *num1*Δ. Linear fits with weights 1/s.e.m., MSAD = 2*D*_MT_Δ*t*+offset are also shown (lines) and the resulting angular diffusion coefficients *D*_MT_ are given in Table 1. Fitting a parabola of a form MSAD = v^2^(Δt)^2^+2*D*_MT_Δ*t*+offset to the data resulted in negative values for v^2^ for all three strains in A and B and thus did not yield a meaningful result.

Live-cell imaging of tubulin-labeled cells revealed that astral microtubules change their orientation with respect to the cell and to the spindle, where one end of the microtubule is attached to the SPB and the other end moves in the cytoplasm or along the cell cortex, thereby sweeping through the cell (Fig. 1, A and B; Movie S1) [5], [13]. To quantify the process of microtubule pivoting, we investigated time series of the angle between the astral microtubule and the spindle in wild-type cells (Fig. 1, B and C). To distinguish whether microtubule pivoting is directed or random, we calculated the mean squared angular displacement (MSAD) [26], and found that it scales roughly linearly with time (Fig. 2, A and B; see also [24]). Such a linear relationship is a feature of random movement, and from the slope we calculated the corresponding effective angular diffusion coefficient of astral microtubules (Table 1).

**Table 1:** Effective angular diffusion coefficients *D*_MT_ of astral microtubules.

Effective angular diffusion coefficient, *D*_MT_ (degrees^2^/s)		wild type	*kar9*Δ	*num1*Δ
MTs from the daughter SPB	*L* = 0.75–1.25 μm	27.3±1.2 (n = 28)	6.8±0.2 (n = 21)	19.8±2.3 (n = 30)
	*L* = 1.25–1.75 μm	28.4±1.6 (n = 8)	6.2±0.3 (n = 20)	19.8±1.7 (n = 22)
MTs from the mother SPB	*L* = 0.75–1.25 μm	13.5±1.6 (n = 13)	7.5±0.7 (n = 14)	6.5±0.6 (n = 10)
	*L* = 1.25–1.75 μm	5.2±1.2 (n = 2)	4.4±0.4 (n = 10)	7.7±1.0 (n = 9)

*D*_MT_ was calculated separately for the microtubules extending from the SPB destined to the daughter cell and from the SPB destined to the mother cell, in each of the 3 strains (wild type, *kar9*Δ, *num1*Δ). *D*_MT_ was obtained by fitting the equation MSAD = 2*D*_MT_Δ*t*+offset to the mean squared angular displacement (MSAD) data shown in Figure 2, with weights 1/s.e.m. The errors are the square root of the diagonal elements of the covariance matrix from the linear fit, n denotes the number of microtubules. The number of cells was 9 for wild type, 10 for *kar9*Δ and 10 for *num1*Δ.

### Kar9 Pathway Increases the Speed of Microtubule Pivoting

To test the role of the Kar9 pathway and the dynein pathway in microtubule pivoting, we analyzed microtubule angles in *kar9*Δ and *num1*Δ cells (examples are shown in Movie S2 and Movie S3, respectively). As in wild type, MSAD of microtubules in *kar9*Δ and *num1*Δ cells scaled linearly with time (Fig. 2, A and B). However, the slope of this relationship, i.e., the angular diffusion coefficient of the microtubules, differed between the strains and between the microtubules extending from the two SPBs. Because the angular diffusion coefficient has been shown to depend on microtubule length [24], in the analysis of the MSAD we used only those microtubules that were 0.75–1.25 μm long.

We first compare the microtubules originating from the daughter-bound SPB in the 3 strains. The angular diffusion coefficient was 4 times lower in *kar9*Δ cells than in wild-type cells (Fig. 2A; Table 1), implying that the Kar9 pathway increases the speed of pivoting of the microtubules extending from the daughter-bound SPB. In *num1*Δ cells, the angular diffusion coefficient was 30% lower than in wild-type cells, suggesting that the dynein pathway also affects microtubule pivoting, though to a smaller extent.

At the mother-bound SPB, the diffusion coefficient in *kar9*Δ and *num1*Δ cells was roughly 2 times lower than in wild-type cells (Fig. 2B; Table 1), suggesting that both the Kar9 and the dynein pathway affect microtubule pivoting in the mother cell. The effect of the Kar9 pathway at the mother-bound SPB is most likely due to the residual amount of Kar9 at this SPB [14].

Finally, we compare the microtubules extending from the two SPBs. In wild-type and *num1*Δ cells, the angular diffusion coefficient of the microtubules originating from the daughter-bound SPB was higher than that of the microtubules extending from the mother-bound SPB, whereas in *kar9*Δ cells such a difference was not observed (Fig. 2, A and B; Table 1). These results are consistent with the finding that Kar9 is preferentially loaded to the daughter-bound SPB and the microtubules emanating from it [14]. Similar results were obtained for longer microtubules (1.25–1.75 μm long; Fig. 2, C and D; Table 1), suggesting that the observed differences in the angular diffusion coefficient are not due to a lack of interaction between the microtubules and the cell cortex because short microtubules were chosen for analysis.

We conclude that the Kar9 pathway has a major effect on the speed of microtubule pivoting. The dynein pathway also influences the speed of pivoting, though to a smaller extent.

### Microtubule Pivoting in Wild-type Cells is an Active Process, Whereas that in Kar9Δ Cells is Consistent with Thermal Motion

Microtubule pivoting may be driven by active components such as motor proteins, or it may be thermally driven. In the latter case, the angular diffusion coefficient of the microtubule decreases with increasing microtubule length [27], [28], [29]. An astral microtubule can be described as a thin stiff rod with one end connected to a fixed point through a free joint, while the other end is free to move [24]. This allows the rod to perform angular movement (scheme in Fig. 1A, dashed lines). Thermally driven angular diffusion of such a rod is described by *D* (degrees^2^/s)  =  (3⋅180^2^ ln(*L*/*d*) *k*_B_ *T*)/(4 π^3^ *L* ^3^ *η*), where *L* is the length and *d* the diameter of the rod, *k*_B_ is the Boltzmann constant, *T* is absolute temperature, and *η* is the viscosity of the medium [27], [28], [29]. This equation is a good approximation for *L*>>*d* [27], [29].

**Figure 3:**
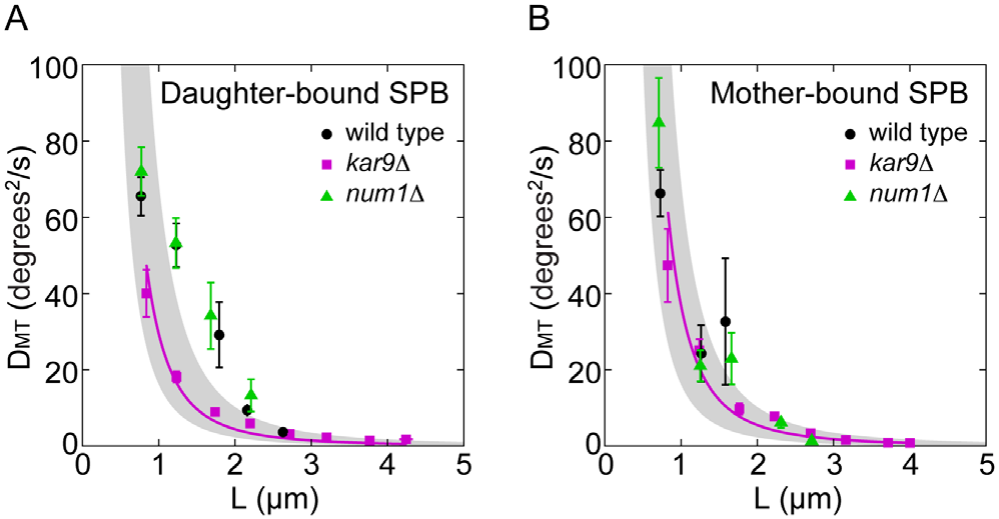
Microtubule pivoting is driven by active processes. The angular diffusion coefficient *D*_MT_ as a function of microtubule length *L*, for microtubules extending from the SPB destined to the daughter cell (A) and from the SPB destined to the mother cell (B). Data from wild-type cells are shown in black, from *kar9*Δ cells in magenta, and from *num1*Δ cells in green. The angular diffusion coefficient was calculated as *D*_MT_ = MSAD/(2Δ*t*), where Δ*t* = 3±0.5 s. MSAD and the corresponding mean microtubule length *L* were calculated from 20-second-long time series of the microtubule angle and length, respectively. The number of data points in each bin was 5–130, with more points for shorter microtubules; error bars represent s.e.m. For *kar9*Δ cells, a single-parameter fit of the equation *D*_MT_ (degrees^2^/s)  =  (3⋅180^2^ ln(*L*/*d*) *k*_B_ *T*)/(4 π^3^ *L* ^3^ *η*) to the data yielded the viscosity *η* = 475 cP in panel A and *η* = 384 cP in panel B (magenta lines). Here, *d* = 25 nm, *k*_B_ = 1.38*10^−23 ^J/K, and *T* = 298.15 K was used. The gray area marks the diffusion coefficient values calculated for a viscosity of 300 cP (upper bound) to 700 cP (lower bound).

We determined the relationship between the length of the astral microtubules and their effective angular diffusion coefficient, which was calculated from the MSAD of the microtubules. We found that the diffusion coefficient decreases with increasing microtubule length in wild-type, *kar9*Δ and *num1*Δ cells, both at the daughter-bound and the mother-bound SPB (Fig. 3, A and B). However, only *kar9*Δ cells showed a decrease consistent with the theoretical prediction for thermally driven motion of a thin rod. The corresponding fit with the effective viscosity of the cytoplasm as a single free parameter suggests that the cytoplasm is roughly 500 times more viscous than water (Fig. 3, magenta lines). This value is of the same order of magnitude as the one previously measured for the cytoplasm of fission yeast cells [30].

The diffusion coefficient in wild-type and *num1*Δ cells did not show a decrease consistent with the theoretical prediction for thermally driven motion of a thin rod. The deviation from this prediction was more pronounced at the daughter-bound than at the mother-bound pole (Fig. 3, A and B). Based on the analysis of the effective angular diffusion coefficient as a function of microtubule length, we conclude that the pivoting of astral microtubules is to a large extent driven by active components, which belong to the Kar9 pathway.

### Kar9 Pathway Promotes Frequent Spindle Movements towards the Bud

**Figure 4:**
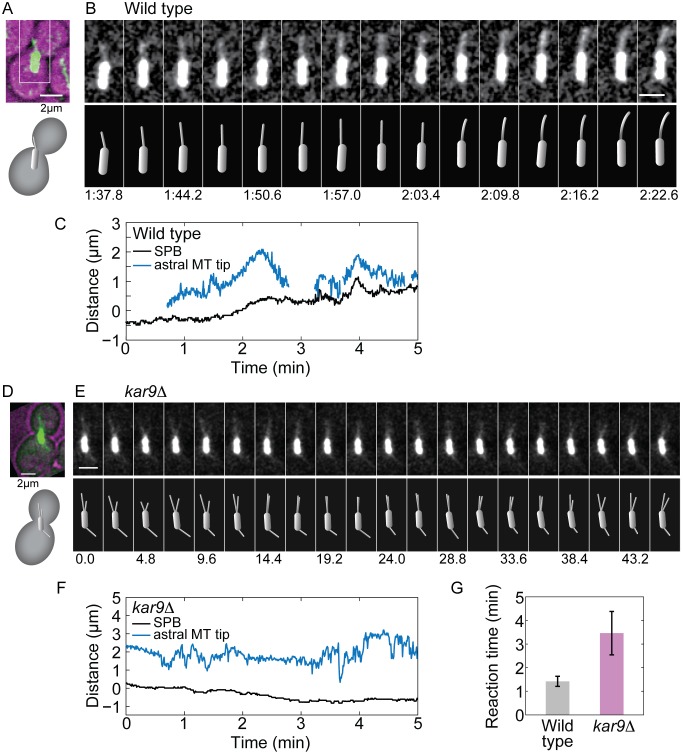
Events of rapid spindle movement are more frequent in cells with an intact Kar9 pathway. (A) Image of the spindle and astral microtubules (green) in a wild-type cell expressing tubulin-GFP (bright-field image of the cell is overlaid in magenta); scale bar is 2 μm. The corresponding scheme is shown below. (B) Time-lapse images of the region marked with the white rectangle in panel A; scale bar is 2 μm. The schemes below show the position of the spindle and astral microtubule in each image; time is given in minutes:seconds. See also Movie S4. (C) Position of the SPB destined to the bud (black) and of the tip of the astral microtubule extending from that SPB (blue) as a function of time, for the cell shown in panels A and B. Position 0 denotes the bud neck. (D)–(F) Data from a *kar9* **Δ** cell; the legend is equivalent to that for panels (A)–(C), respectively. Time is given in seconds. See also Movie S5. (G) Reaction time for wild-type and *kar9*Δ cells. Number of cells is 16 in wild type and 15 in *kar9*Δ; number of astral microtubules in the daughter cell is 50 in wild type and 17 in *kar9*Δ; number of events is 29 for wild type and 12 for *kar9*Δ; total observation time is 350 min for wild type and 239 min for *kar9*Δ. Error bars represent s.e.m.; *p* = 0.05 from a two-sided t-test comparing the reaction time in wild-type and *kar9*Δ cells.

The transition of one spindle pole from the mother into the daughter cell does not occur in a smooth and continuous manner but exhibits phases of rapid movements towards the daughter cell, which are sometimes followed by similar retractions (Fig. 4, A–C; Movie S4). These events can be translation or rotation or a combination of both. We hypothesized that the pivoting of the astral microtubules around the spindle pole, which results in a sweeping movement of the microtubule inside the daughter cell, helps them find cortical anchors for dynein. To test this hypothesis, we measured the time elapsed from the entry of an astral microtubule into the daughter cell until the beginning of a pulling event, in which the spindle moves towards the daughter cell. We refer to this time as reaction time. When a microtubule enters the daughter cell and the spindle subsequently exhibits an event of rapid motion towards the daughter cell, this suggests that the microtubule has found a cortical anchor site for dynein, which then pulls on the microtubule.

We found that in *kar9*Δ cells astral microtubules often remained in the daughter cell for a long time, without an accompanying movement of the spindle towards the daughter (Fig. 4, D–F; Movie S5). Consistently, the mean reaction time of the spindle was longer in *kar9*Δ cells than in wild-type cells (Fig. 4G). In both strains, the reaction time was correlated neither with the distance between the daughter-bound SPB and the neck (*r* ^2^ =  −0.17 and 0.001 for wild type and *kar9*Δ, respectively), nor with the length of the astral microtubules (*r* ^2^ = 0.33 and −0.08 for wild type and *kar9*Δ, respectively). These results argue against the possibility that the longer reaction time in *kar9*Δ cells is a consequence of a larger average distance between the daughter-bound SPB and the neck or longer microtubules than in wild type. Although we cannot exclude the possibility that *kar9*Δ cells have a longer reaction time because of other defects in this mutant, we favor the model in which the microtubules in *kar9*Δ cells show this delay because they pivot less than in wild type. We propose that the Kar9 pathway, by increasing the speed of microtubule pivoting, helps the microtubules to explore the space laterally. This angular movement of the microtubules improves their search for cortical anchor sites for dynein in the daughter cell, which results in pulling of the spindle from the mother to the daughter cell.

## Discussion

### The Mechanism of Microtubule Pivoting

During mitosis in budding yeast, the spindle forms inside the mother cell and has to move through a narrow neck into the daughter cell or the bud. Two types of motor proteins are involved in the generation of force on astral microtubules and thus on the spindle: myosin V, which binds to the plus end of the microtubule via the adaptor protein Kar9, and cytoplasmic dynein [6], [7], [10], [11]. We have shown that in the absence of Kar9, microtubules pivot substantially slower than in wild type. Thus, the pivoting motion of astral microtubules is most likely driven by the movement of myosin along cortical actin filaments. As the plus end of the microtubule is being moved by myosin, while the minus end is bound to the SPB, which does not move as much as the plus end, the whole astral microtubule pivots around the SPB. Our observation that the microtubules extending from the daughter-bound SPB pivot faster than those at the mother-bound SPB is consistent with the fact that Kar9 is predominantly found at the daughter-bound SPB and the associated microtubules [14].

Similarly to myosin, cortically anchored dynein in the daughter and the mother cell pulls on astral microtubules and thus on the spindle, thereby inducing their angular motion. In *kar9*Δ cells, the observed less extensive pivoting of astral microtubules is most likely due to a combination of dynein-driven motion and thermal motion of microtubules that are not in contact with the cell cortex.

In addition to the forces acting on the plus end of the microtubule, the events at the minus end are important for the pivoting motion. In principle, pivoting is possible if one end of the microtubule is freely jointed to a fixed point, which allows the microtubule to rotate around this point. In the context of budding yeast mitosis, the minus ends of astral microtubules are freely jointed to the SPB. It will be interesting to unravel the molecular basis and the physical properties of this free joint that allows for pivoting of the microtubules.

### Dual Role of Microtubule Pivoting in Spindle Orientation and Movement

While the spindle is in the mother cell, myosin moves the plus end of astral microtubules along actin cables, which are polarized towards the neck, thereby directing the overall pivoting of astral microtubules and the movement of the spindle towards the neck. When the spindle reaches the neck, astral microtubules enter the daughter cell. To pull the spindle through the neck, dynein that is on the microtubules needs to be off-loaded to the cortical anchor protein Num1 [22].

**Figure 5:**
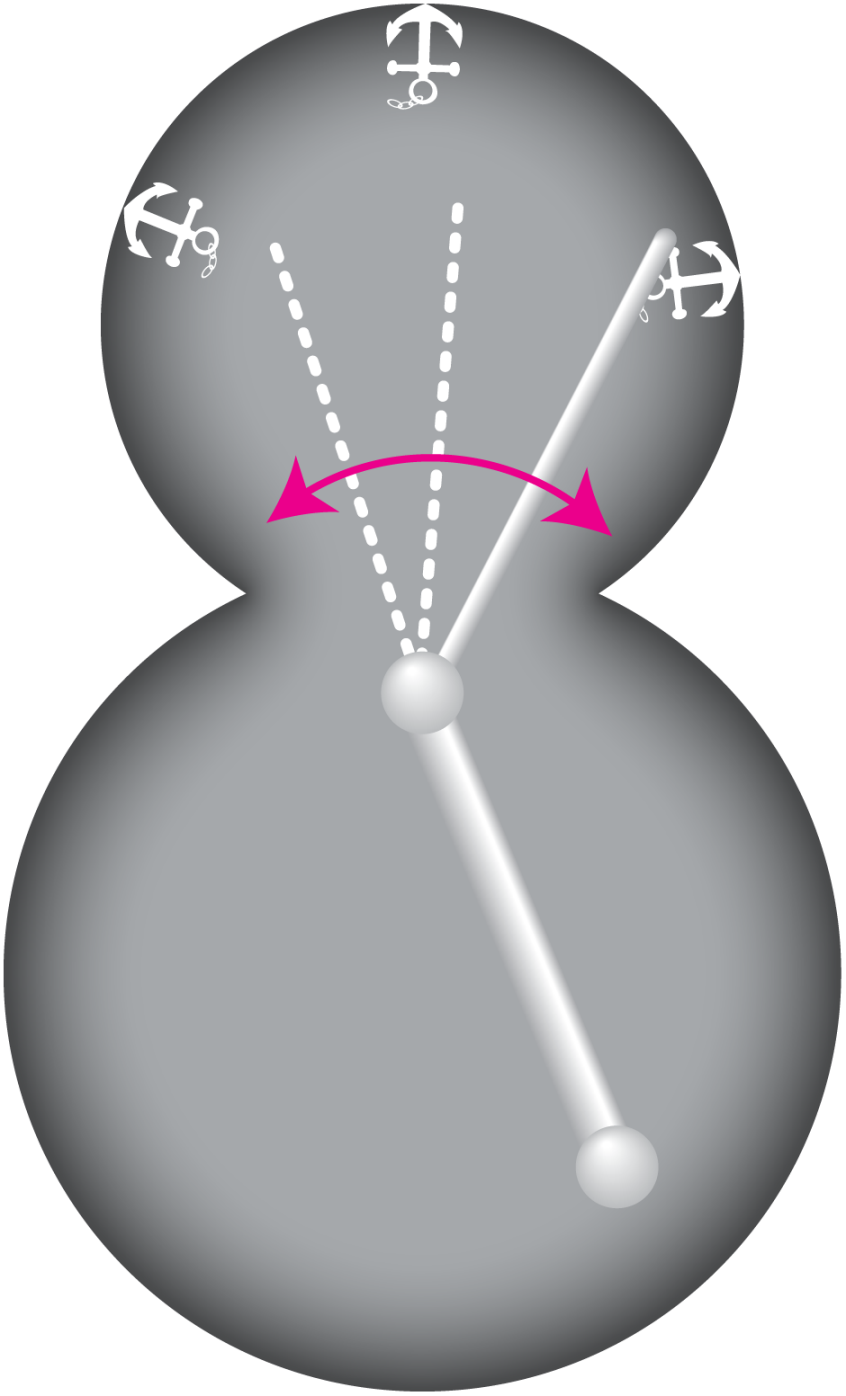
The pivoting motion of astral microtubules helps them to find cortical anchors. When astral microtubules enter the daughter cell, they exhibit pivoting motion around the SPB. This motion may serve as a search mechanism for cortical anchors for dynein. Once the microtubule finds an anchor, dynein can be off-loaded and the spindle is pulled towards the daughter cell.

We have shown here that astral microtubule pivoting, which is driven mainly by the Kar9 pathway, reduces the time needed for an astral microtubule inside the daughter cell to generate pulling of the spindle towards or into the daughter cell. We interpret these pulling events as successful microtubule explorations of the cortex in search for the anchor sites, followed by dynein off-loading to the anchor and pulling on the microtubule. Thus, pivoting of microtubules has two roles. First, it helps to orient the spindle towards the neck inside the mother cell. Second, pivoting facilitates cortical capture of microtubules inside the daughter cell, which is needed for dynein to become off-loaded from the microtubule to the cortical anchors in order to exert force on the microtubule (Fig. 5).

### Pivoting as a Search Strategy

We found that at short time-scales, microtubules pivot around the spindle pole in a random manner, thereby exploring the space through which they move. We propose that the pivoting movement of microtubules facilitates their search for cortical anchors. This search mechanism is similar to the one used by microtubules in order to find kinetochores during mitosis in fission yeast, which is also based on microtubule pivoting around the SPB [24], [31]. However, while the pivoting motion of the polar microtubules inside the fission yeast nucleus is driven by thermal forces [24], the pivoting motion of astral microtubules in the budding yeast cytoplasm is driven largely by myosin motors. In both cases, irrespective of whether the pivoting is a passive or an active process, this motion allows microtubules to explore space laterally, as they search for targets such as kinetochores or cortical anchors.

## Materials and Methods

### Sample Preparation

The strains used in this work are listed in Table S3. Cells were grown on agar plates with appropriate supplements and stored in a cold room at 4°C. For experiments, cells were transferred to liquid medium and stored overnight in a shaker at 25°C in order to prepare log-phase cultures. Samples for experiments were prepared by using 35 mm MatTek plastic dishes (MatTek Corporation, Ashland, MA, USA). First, a glass coverslip was glued from the inside of the dish onto the hole on the bottom by using silicon grease. The dish was turned upside down and the hole was filled with melted agar and covered with a second coverslip. After the agar solidified the second coverslip was removed and 5–8 μl of the liquid cell culture was pipetted onto the thin agar patch on the bottom of the dish. When the liquid medium had dried out the sample was closed with a new high precision 0.17±0.005 mm coverslip (Menzel Glaeser, Glasbearbeitungswerk GmbH & Co. KG, Braunschweig, Germany) and sealed with silicon grease to prevent drying out.

### Microscopy

Microscopy was performed on an Andor spinning disc microscope (Andor Technology, Belfast UK). The scan head was a Yokagawa CSU-X1 unit with a pinhole size of 24 μm and a pinhole spacing of 240 μm. An Olympus UplanSApo 100x/1.4 N.A. oil objective (Olympus, Tokyo, Japan) and an Andor iXon EM+ DU-897 BV cooled and back illuminated Electron Multiplying Charge Coupled Device (EMCCD) camera with a physical pixel size of 16 μm (Andor Technology plc., Belfast, UK) were used. The resulting xy-pixel size in the images was 77.4 nm. For excitation, a Sapphire 488 nm solid-state laser was used (75 mW; Coherent, Inc., Santa Clara, CA, USA). The exposure time was 40–70 ms. The microscope was equipped with a motorized Prior ProScanIII xy scanning stage (Prior Scientific Inc., Rockland, MA, USA) together with a Prior NanoScanZ piezo inset to control the z-position.

To image the fast angular motion of the astral microtubules, z-stacks of images were acquired with a time resolution of 0.4–0.6 s. This was achieved by reducing the imaging area to the observed cell only. To ensure that the entire spindle and astral microtubules were captured, z-stacks of 4–6 optical slices with a z-spacing of 300–600 nm were acquired. A bright-field image of the cell was acquired immediately before the start of the fluorescence imaging in order to visualize the outline of the cell. A single bright-field image was sufficient because the cells did not move.

### Image Processing

Image processing was performed with the open-source Fiji software package [32]. Maximum-intensity projections were created from each z-stack and used for the measurements of the position of the spindle and astral microtubules. We did not measure positions along the z-axis because the corresponding point-spread function of the microscope is about 800 nm, which is more than half of the length of a typical microtubule. We have previously estimated the systematic error resulting from two-dimensional measurements of microtubule length in maximum-intensity projections, compared to three-dimensional measurements in z-stacks, see Supplementary Note 3 in Ref. [24]. If one assumes that microtubules extend from the SPB isotropically, the real microtubule length is roughly 20% larger than the average measured microtubule length, whereas the real angular diffusion coefficient of the microtubules is roughly 30% smaller than the measured one [24]. In the current work, the corrections are most likely smaller because the mother-bud axis of the imaged cells lies roughly parallel to the imaging plane. Thus, the spindle and the astral microtubules are more often found at small than at large angles with respect to the imaging plane, which implies that the measured lengths and angles are closer to the real ones than in the estimate mentioned above. In addition, this systematic error affects the angular diffusion coefficient of the astral microtubules emanating from the daughter-bound and the mother-bound SPB in a similar man Proper ner. Based on these arguments, we conclude that the observed differences in the measured angular diffusion coefficients are unlikely due to the two-dimensional measurements of microtubule length and angle.

To automatize the tracking of the mitotic spindle, a new custom-made Fiji plugin was developed and implemented together with Johannes Schindelin (Image Processing Facility, MPI-CBG). When the spindle is in the mother cell, it is usually short (<3 μm long) and straight, thus it was modeled as a straight line. As initialization of the tracking, a straight line was manually drawn along the spindle. In order to reduce the noise, the line width was set to 4 pixels. Before automatically adjusting the drawn line to the spindle, the average intensity of the image was subtracted as background and the image was smoothed by a Gaussian blur of 1–2 pixels radius. The adjustment algorithm involves two steps: (i) The manually drawn initialization line is rotated until it is aligned to the intensity profile of the spindle. For this purpose line profiles perpendicular to the initial line at its endpoints were taken. To those profiles a Gaussian function was fitted to determine the central point of the intensity profile of the spindle. The central points of the two line profiles were used to calculate the line of the spindle. (ii) The ends of this line were adjusted to the spindle ends by inspecting the intensity profile along the line. The endpoints were defined where the intensity drops to half the maximum value. This two-step algorithm was repeated until convergence and the resulting line was used as initialization for the next frame. The resulting lines corresponding to the spindle position were visually inspected and their position was adjusted manually in cases of error. The coordinates of the spindle poles were exported as a text file for further analysis.

Due to the fast angular movement, growth and shrinkage of astral microtubules, in combination with their dim signal, it was not possible to extend the tracking algorithm described above to astral microtubules. These microtubules were therefore tracked manually by clicking on their tip in the maximum-intensity projection. The measured coordinates were obtained by using the manual tracking plugin for Fiji and stored in a text file. In addition to the coordinates of the spindle poles and astral microtubules, the position of the mother-daughter neck and the most distant point of the daughter cell were measured in a single bright-field image of each cell.

### Angles and Distances

Data analysis and plotting was done using the open-source R programming language [33]. Coordinates of the spindle poles and microtubule tips were tracked over time in the maximum intensity projections as described above. The length of the spindle and astral microtubules was calculated from the tracked coordinates of the spindle poles and astral microtubule tips. Furthermore, the distance from the spindle poles and the astral microtubule tips associated with the daughter spindle pole to the neck were calculated. The distance from an object to the neck was denoted negative for objects in the mother cell and positive for those in the daughter cell.

The angles α of the astral microtubules were calculated with respect to the spindle axis, and were defined in direction away from the spindle. The spindle axis was calculated with respect to the cell axis towards the daughter cell. Thus, α lies within the range [−180°, +180°].

### Detection of Events of Rapid Spindle Pole Movement

Events of rapid spindle movement were detected manually in the following way. Cases where the spindle is completely in the mother cell and astral microtubules have not entered the daughter cell yet were selected. In these cases, the time of the microtubule entry into the daughter cell was recorded, as well as the time of the subsequent reaction of the spindle, which was either rapid translation towards the neck or rotation towards the neck. The time difference between microtubule entry and the spindle reaction was denoted as reaction time.

## Supporting Information

Table S1 **Spindle translocation efficiency, cell and spindle size.**
(DOC)

Table S2 **Astral microtubule lifetime and length.**
(DOC)

Table S3 **Strains used in this study.**
(DOC)

Movie S1 **Dynamics of the spindle and astral microtubules in a wild-type cell.** Live-cell microscopy of a budding yeast cell during mitosis. Microtubules are labeled with GFP-TUB1. The spindle is visible as a thick bright line in the center and astral microtubules grow outwards. Note that astral microtubules perform pivoting motion around the SPB. At the beginning of the movie, the astral microtubule extending from the daughter-bound (upper) SPB and the one at the mother-bound (lower) SPB perform extensive rotations around the SPB, while later in the movie the microtubule at the daughter-bound SPB rotates more than the one at the mother-bound SPB. Images were acquired at 0.4-second intervals, represent maximum-intensity projections of 5 slices per time frame, and are displayed at 20 frames per second. The scale bar represents 2 μm. Time is given in minutes:seconds. The movie corresponds to Figure 1B.
(MOV)

Movie S2 **Dynamics of the spindle and astral microtubules in a** ***kar9*** **Δ cell.** Astral microtubules in this cell are significantly longer than in wild type and show less pivoting. In the beginning of the movie, the spindle is roughly perpendicular to the mother-bud axis. Microtubules are labeled with GFP-TUB1. Images were acquired as in Movie S1. The scale bar represents 2 μm. Time is given in minutes:seconds.
(MOV)

Movie S3 **Dynamics of the spindle and astral microtubules in a** ***num1*** **Δ cell.** Microtubules show pivoting motion as in wild type, but the spindle does not enter the bud. Microtubules are labeled with GFP-TUB1. Images were acquired as in Movie S1. The scale bar represents 2 μm. Time is given in minutes:seconds.
(MOV)

Movie S4 **Microtubule pivoting and spindle translocation in a wild-type cell.** Spindle translocation into the bud occurs between 1∶40 and 2∶30 minutes. Microtubules are labeled with GFP-TUB1. Images were acquired as in Movie S1. The scale bar represents 2 μm. Time is given in minutes:seconds. The movie corresponds to Figure 4B.
(MOV)

Movie S5 **A** ***kar9*** **Δ cell without spindle translocation into the bud.** Microtubules are labeled with GFP-TUB1. Images were acquired as in Movie S1. The scale bar represents 2 μm. Time is given in minutes:seconds. The movie corresponds to Figure 4E.
(MOV)
