# Supplementary material for: Astral Microtubule Pivoting Promotes Their Search for Cortical Anchor Sites during Mitosis in Budding Yeast
Source: PLoS One. 2014 Apr 10;9(4):e93781. doi: 10.1371/journal.pone.0093781 (PMC3983083; doi:10.1371/journal.pone.0093781)
Supplement: Table S1 — Spindle translocation efficiency, cell and spindle size. (DOC) [file pone.0093781.s001.doc]

**Table S1: Spindle translocation efficiency, cell and spindle size.**

|  | *wild type* | *kar9*Δ | *num1*Δ |
| --- | --- | --- | --- |
| Successful translocations of the spindle into the daughter cell | 49/50 | 50/50 | 40/50 |
| Time elapsed from the moment when the spindle reached a length of 2 µm until spindle entry into the daughter cell | 150±25 s | 261±39 s* | 146±49 s |
| Diameter of the mother cell | 5.3±0.1 µm | 6.2±0.1 µm** | 6.1±0.2 µm** |
| Spindle length at entry into the daughter cell | 3.5±0.2 µm | 4.0±0.3 µm | 3.4±0.3 µm |

Values are reported as mean±s.e.m; 50 cells were observed in each strain; * 0.005<*p*<0.05, ** *p*<0.005 from a t-test comparing mutants with wild type. Note that the time of spindle entry into the daughter cell (second row) was measured from the moment when the spindle reached a length of 2 µm and thus cannot be compared with the time of entry in Figures 1 and 4 and the movies, where time 0 denotes the beginning of imaging.
