# Supplementary material for: Astral Microtubule Pivoting Promotes Their Search for Cortical Anchor Sites during Mitosis in Budding Yeast
Source: PLoS One. 2014 Apr 10;9(4):e93781. doi: 10.1371/journal.pone.0093781 (PMC3983083; doi:10.1371/journal.pone.0093781)
Supplement: Table S2 — Astral microtubule lifetime and length. (DOC) [file pone.0093781.s002.doc]

**Table S2: Astral microtubule lifetime and length.**

|  | *wild type* | *kar9*Δ | *num1*Δ |
| --- | --- | --- | --- |
| Lifetime of astral microtubules extending from the *daughter* spindle pole | 72.6±8.6 s (n=71) | 212.0±32.3 s (n=43) | 110.8±14.7 s (n=57) |
| Lifetime of astral microtubules extending from the *mother* spindle pole | 49.0±6.3 s (n=60) | 263.0±52.6 s (n=25) | 69.6±9.0 s (n=60) |
| Length of astral microtubules extending from the *daughter* spindle pole | 1.1±0.05 µm (n=71) | 1.9±0.05 µm (n=43) ** | 1.1±0.03 µm (n=57) |
| Length of astral microtubules extending from the *mother* spindle pole | 0.9±0.03 µm (n=60) | 1.7±0.05 µm (n=25) ** | 1.0±0.04 µm (n=60)* |

Values are reported as mean±s.e.m, *n* denotes the number of microtubules. To calculate the typical microtubule length, time-series of microtubule length were divided into non-overlapping 20-second-long intervals and for each interval the mean length was calculated. * 0.005<*p*<0.05, ** *p*<0.005 from a t-test comparing mutants with wild type.
