# Supplementary material for: Astral Microtubule Pivoting Promotes Their Search for Cortical Anchor Sites during Mitosis in Budding Yeast
Source: PLoS One. 2014 Apr 10;9(4):e93781. doi: 10.1371/journal.pone.0093781 (PMC3983083; doi:10.1371/journal.pone.0093781)
Supplement: Table S3 — Strains used in this study. (DOC) [file pone.0093781.s003.doc]

**Table S3: Strains used in this study.**

| Strain | Genotype | Source |
| --- | --- | --- |
| yJC5919 (wild type) | *MATα ura3–52 lys2–801 leu2-*Δ*1::GFP-TUB1::LEU2 his3-*Δ*200 trp1-*Δ*63* | Jeff Moore, Cooper lab, St. Louis, USA |
| KBY5058 (*kar9*∆) | *MATa* *trp1Î leu2∆ his3∆ lys2∆ ura3∆ kar9∆ LEU2 Tub1GFP:Ura3 (pAFS125)* | Elaine Yeh, Bloom Lab, Chapel Hill, USA |
| yJC5918 (*num1*∆) | *MATa* *num1*∆*::HIS3* *ura3–52 lys2–80 leu2-*Δ*1::GFP-TUB1::LEU2 his3-*Δ*200 trp1-*Δ*63* | Jeff Moore, Cooper lab, St. Louis, USA |
